# Natural history and predictors for progression in pediatric keratoconus

**DOI:** 10.1038/s41598-023-32176-5

**Published:** 2023-03-27

**Authors:** Rosalia Antunes-Foschini, Henrique Doná, Pedro Henrique Sant’Anna de Mello, Renato Bredariol Pereira, Isadora Mendes Marqueis, Eduardo Melani Rocha, Sidney Julio de Faria-e-Sousa, Gleici Castro Perdona

**Affiliations:** 1 Department of Ophthalmology, Otorhinolaryngology, and Head and Neck Surgery, Ribeirão Preto Medical School, University of São Paulo, Av. Bandeirantes, 3900, Ribeirão Preto, SP CEP 14049-900 Brazil; 2 Department of Ophthalmology, Otorhinolaryngology, and Head and Neck Surgery, Ribeirão Preto Medical School, University of São Paulo, Ribeirão Preto, SP Brazil; 3 Department of Social Medicine, Ribeirão Preto Medical School, University of São Paulo, Ribeirão Preto, SP Brazil

**Keywords:** Epidemiology, Paediatric research

## Abstract

We studied the demographic and clinical predictors associated with keratoconus progression in a pediatric population. Retrospective cohort study. We evaluated 305 eyes without previous surgeries from 168 patients, 9 to < 18 years old, and with a minimum 36-month follow-up in a hospital corneal ambulatory. We used Kaplan-Meyer survival curves; the dependent variable (main outcome measure) was the interval time (months) until the event, defined as an increase of 1.5 D in the maximum keratometry (Kmax), obtained with Pentacam. We evaluated the predictors: age (< or ≥ 14 years), sex, keratoconus familial history, allergy medical history, and the baseline tomographic parameters: mean keratometry (Km), Kmax (< or ≥ 55 D); and thinnest pachymetry (TP). We used log-rank tests and compared median survival times for right (RE)/left eyes (LE) and better (BE)/worse eyes (WE). A *p* value < 0.05 was considered significant. The patients’ mean ± SD age was 15.1 ± 2.3 years old; 67% were boys, 30% were < 14 years, 15% had keratoconus familial history, and 70% were allergic. The general Kaplan-Meyer curves showed no differences between RE/LE or BE/WE. RE with allergy and LE with Kmax ≥ 55 D had smaller survival times ((95%CI 9.67–32.1), p 0.031 and (95%CI 10.1–44.1), *p* 0.042, respectively). For BE and WE, Kmax ≥ 55 D had smaller survival times ((95% CI 6.42- ), *p* 0.031 and (95%CI 8.75–31.8), *p* 0.043, respectively). Keratoconus progression was similar between RE/LE and BE/WE. Steepest corneas are predictors of faster progression. Allergy is also a predictor of keratoconus progression in RE.

## Introduction

Keratoconus is a progressive corneal ectasia characterized by progressive thinning and protrusion of the cornea^1,2^, with a prevalence of 0.4–86 cases per 100,000 individuals^3^. Its progression is more aggressive at puberty^4–6^, and interferes greatly with their quality of life^7^. However, even more aggressive, the KERALINK study showed that 57% of children aged younger than 17 years old did not show progression after 18 months^8^.

The current recommended treatment for halting the progression of keratoconus and preventing visual loss is crosslinking (CXL), an ultraviolet A (UVA) light therapy associated with riboflavin eye drops^9^.

The definition of keratoconus progression is challenging, and published literature considers changes in topographic data, manifest refraction, and visual acuity, among other parameters. However, most of the data in pediatric keratoconus described an increase of at least 1.0 D in the maximum keratometry (Kmax) for CXL indication^10–16^. Chatzis et al.^4^ recommended CXL even without documented progression. However, there are concerns about performing CXL in patients with best-corrected visual acuity equal to logMAR 0 due to the possibility of haze^17^ or other surgical acute or long-term complications. Despite this, good visual acuity is not mentioned as an exclusion criterion in any study.

There is a need to identify the predictors associated with keratoconus progression and, therefore, the possibility of planning the CXL procedure. This study aimed to look for some of these predictors in a pediatric population.

## Patients and methods

This was a retrospective cohort study to evaluate the progression of keratoconus in pediatric patients of both sexes, aged nine to younger than 18 years old, who were under the care of a corneal ambulatory clinic in Hospital das Clínicas, Ribeirão Preto Medical School, University of São Paulo. In this age group, the appointments are usually scheduled every six months and may be up to every three months, depending on age or disease severity. Patients initially undergo tomographic exams on the day of the appointment, followed by a medical evaluation. This study was approved by the Ethics Committee in Human Research at Ribeirão Preto General Hospital (approval number 61891816.6.0000.5440), which waived the informed consent due to the retrospective nature of the study, and followed the tenets of the Declaration of Helsinki.

In this study, the dependent variable was defined as the increase of at least 1.5 D in the maximum keratometry (Kmax) or the occurrence of acute corneal hydrops. The time to event was defined as the time between the date of the first appointment with good QS tomography and the interval time between the date the progression was detected and the one immediately before (interval-censored time). In case the first tomography had impaired QS in the frontal surface, the date of the next one with QS OK was considered. Left censored patients (subjects with only one appointment during the period or if all their tomographic exams showed impaired QS on the frontal surfaces) were excluded from the analysis. The eyes were middle censored if they were submitted to any ocular surgery (CXL, intracorneal ring implantation, or corneal transplant) without detecting progression in the previous period or if they lost clinical follow-up.

**Figure 1:**
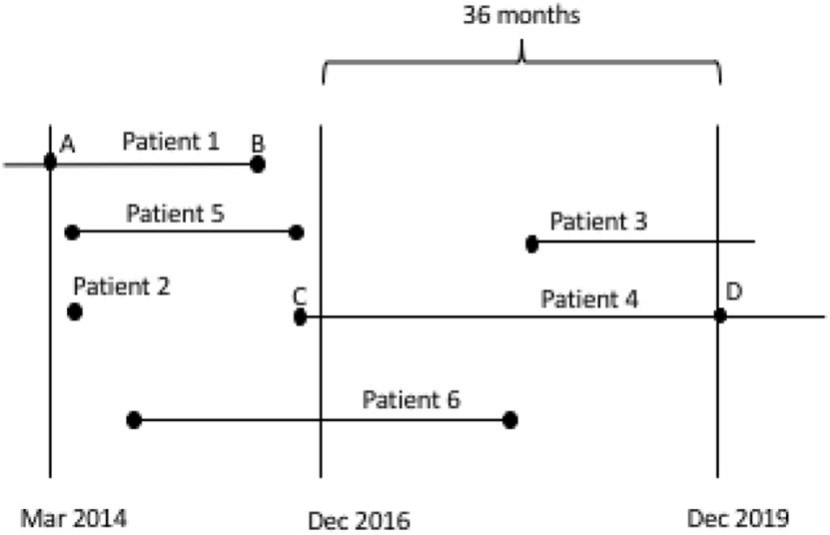
Examples of patients who were or were not included in this survival analysis. Patient 1: analyzed from time points A (T0) to B; (event or censoring) Patient 2: censored after the first appointment; Patient 3: excluded due to not having the possibility of being evaluated during a minimum period of at least 36 months; Patient 4: analyzed from time points C (T0) to D (censoring); Patients 5 and 6: included.

The patients’ records, and their tomographic exams, were obtained from all subjects with a follow-up of at least 36 months, within a period of 70 months (March 2014–December 2019 (Fig. 1). Clinical signs of keratoconus and corneal tomography confirmed the diagnosis based on the global consensus on keratoconus and ectatic diseases^7^.

Corneal imaging was obtained using the principle of Scheimpflug (Pentacam HR, Oculus Optikgeräte GmbH, Wetzlar, Germany), with the images captured in the automatic mode, in a dark room, by an experienced technician. Corneal tomographic parameters, specifically Km (mean) and Kmax (maximum) power of the anterior sagittal power map in a 3 mm zone around the corneal apex as well as the thinnest pachymetry (TP), defined as the smallest measured corneal thickness, were obtained from their exams. The pachymetric data with anterior surface QS scored as "OK" but associated with posterior surface QS marked as "yellow" or "red" were not evaluated either.

The patients who suffered a surgical procedure in only one eye had the other eye analyzed. The right eyes (RE) and the left eyes (LE) were evaluated separately, with a survival curve for each one. As keratoconus is a very asymmetric condition between both eyes, contralateral eyes with normal tomographies or forme fruste keratoconus were also included. Better (BE) and worse eyes (WE) survival curves were also analyzed (In this analysis, only patients who had both eyes evaluated were included.) For the BE and WE classification, the one with the highest Km was considered the worse.

Data on the diagnosis of ocular allergy, allergic rhinitis, asthma, and familial history of keratoconus (confirmed diagnosis in our hospital or strongly suggestive history of surgical procedures related to keratoconus in the relatives) were also collected from their medical records.

**Figure 2:**
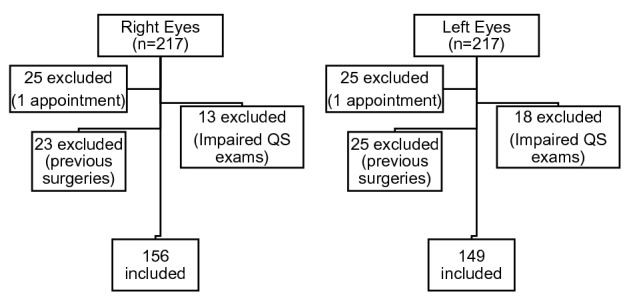
Pediatric keratoconus: Distribution of the eyes, showing the reasons for exclusion and the final number included.

Patients who had only one appointment during the 36 months-period, the ones with previous surgeries (corneal transplant, intrastromal rings, or CXL) in both eyes, and the ones whose all of the exams had abnormal anterior surface quality specifications (QS) provided by the manufacturer, (marked as "yellow" or "red") were excluded (Fig. 2). Other exclusion criteria were ocular malformations, and previous ocular trauma or herpetic keratitis in that specific eye.

We looked for the following potential predictors: age (< 14, or ≥ 14 years old); sex (male, female), familial history of keratoconus, positive medical history of allergy (allergic conjunctivitis, and/or allergic rhinitis, and/or asthma, and/or atopic dermatitis), and the following tomographic parameters: mean keratometry (Km), divided into four groups, based on ABCD grading system^18^ (less than 48 D, between 48 and 53 D, between 53 and 55 D, and equal or greater than 55 D); maximum keratometry (Kmax), divided into two groups (less than 55 D, and equal or greater than 55 D); and TP, divided into four groups, based on ABCD grading system^18^ (equal or greater than 490 μm, between 490 and 450 μm, between 450 and 400 μm, and equal or less than 400 μm). We also collected data from presenting^18^ or distant best-corrected visual acuity with glasses, taken at the time they entered the study.

The datasets analyzed during the current study are available from the corresponding author on reasonable request. All statistical analyses were performed using the statistical analysis system R: Core Team, Vienna, Austria^19^. Frequency tables were used for descriptive analysis. We used the log-rank test and considered interval time^20^ until the occurrence of the event. We compared the median survival times for the RE and the LE; and for the BE and the WE. A *p* value of less than 0.05 was considered to be statistically significant.

## Results

This study evaluated the natural history of keratoconus in a Brazilian pediatric cohort, using survival curves as the statistical analysis, with a minimum follow-up of 36 months.

Figure 2 shows the distribution of 434 eyes from 217 subjects, which resulted in the analysis of 156 RE and 149 LE from 168 patients. Their mean ± SD age was 15.1 ± 2.3 years when they entered the study. Twenty-five patients were excluded due to only one appointment in the 36 month-period, and 21 patients due to previous surgery in both eyes, surgery in one eye and impaired tomography in the other, or impaired tomography in both eyes.

Table 1 shows demographic data in the study group of 168 individuals.

**Table 1:** Pediatric keratoconus: demographic and medical data in a cohort study with 305 eyes of 168 individuals.

N = 305		N
Sex	Female	102 (33%)
	Male	203 (67%)
Age	< 14 y	91 (30%)
	≥ 14 y	214 (70%)
Familial history	no	258 (85%)
	Yes	47 (15%)
Allergy	No	91 (30%)
	yes	214 (70%)

Supplemental Tables 1 and 2 shows the tomographic data distributions (Km, Kmax, and TP severities) among RE and LE and BE and WE. Supplemental Table 3 summarizes the baseline tomographic data and the baseline visual acuity data of the four groups, and the total sample.

Supplemental Fig. 1 shows data about allergic medical history, and familial history collected from the patients’ medical records.

### Survival time for the right and left eyes

**Figure 3:**
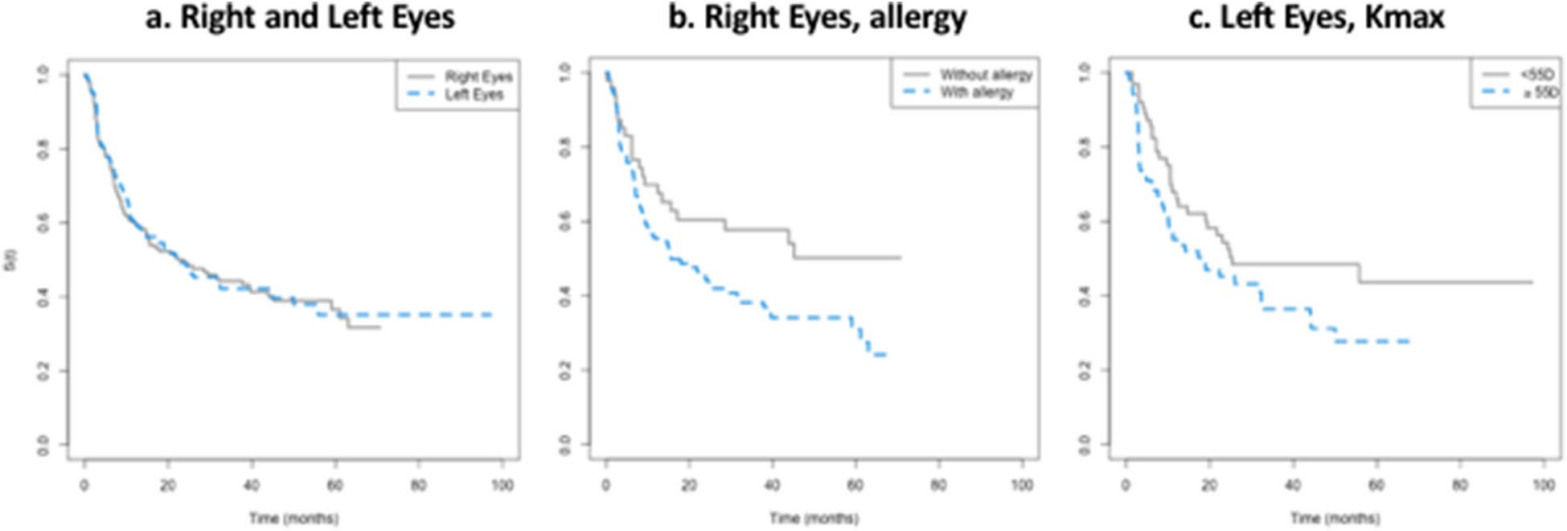
Kaplan Meyer empirical curves of time until progression, in months. (**a**) Right and left eyes, overall progression (Right Eyes (n = 156); 88 events; Median of 21.9 CI95% (14.5; 39.7). Left Eyes (n = 149); 76 events; Median of 22.4 CI95% (13.4; 50.0). *p* value = 0.76); (**b**) Right eyes, by the presence of allergy (*p* value 0.032); and (**c**). Left eyes, by Kmax severity (*p* value = 0.043).

The median survival time to the event did not differ by RE or LE (Fig. 3a). The survival time for an approximately 12-month period was 0.59 (95%CI 0.52–0.68) for the RE and 0.60 (95%CI 0.52–0.69) for the LE, meaning that approximately 40% of both RE and LE had the event. Only two right eyes suffered acute corneal hydrops during the whole 36-month period.

Concerning the predictors, the RE from patients with allergy (Fig. 3b), and the LE with Kmax ≥ 55 D had smaller survival time (faster progression) (Fig. 3c). Supplemental Table 4 summarizes RE and LE statistical data.

### Survival time for the better and worse eyes

**Figure 4:**
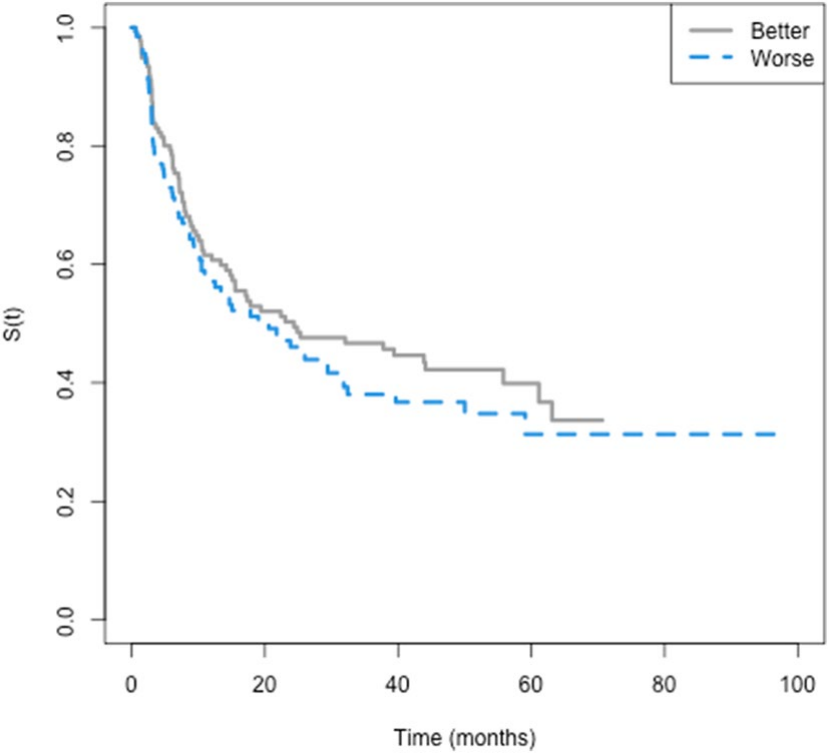
Better and worse eyes: Kaplan Meyer empirical curves of time until progression, in months. The eye with the highest Km between both eyes was considered the worse eye. Better (n = 136); 72 events; Median of 24.9 CI95% (15.5, 61.1). Worse (n = 136); 77 events; Median of 19.0; CI95% (11.0, 31.8). *p* value = 0.301.

This analysis was performed in 272 eyes of 136 patients. The worse eyes were compounded by 73 (53.7%) left eyes. Among 136 patients, 47 (35%) had both eyes with baseline Kmax ≥ 55 D. The median survival time to the event did not differ by BE and WE. Figure 4 shows a vertical line that points out ten months to 0.62 of survival. From this time point on, the survival curves become distinct, although without statistical significance. The survival times for an approximately 12-month period were 0.61 (95%CI 0.53–0.70) and 0.56 (95%CI 0.48–0.66) for the BE and WE, respectively, with events occurring in 39 and 44% of the individuals.

**Figure 5:**
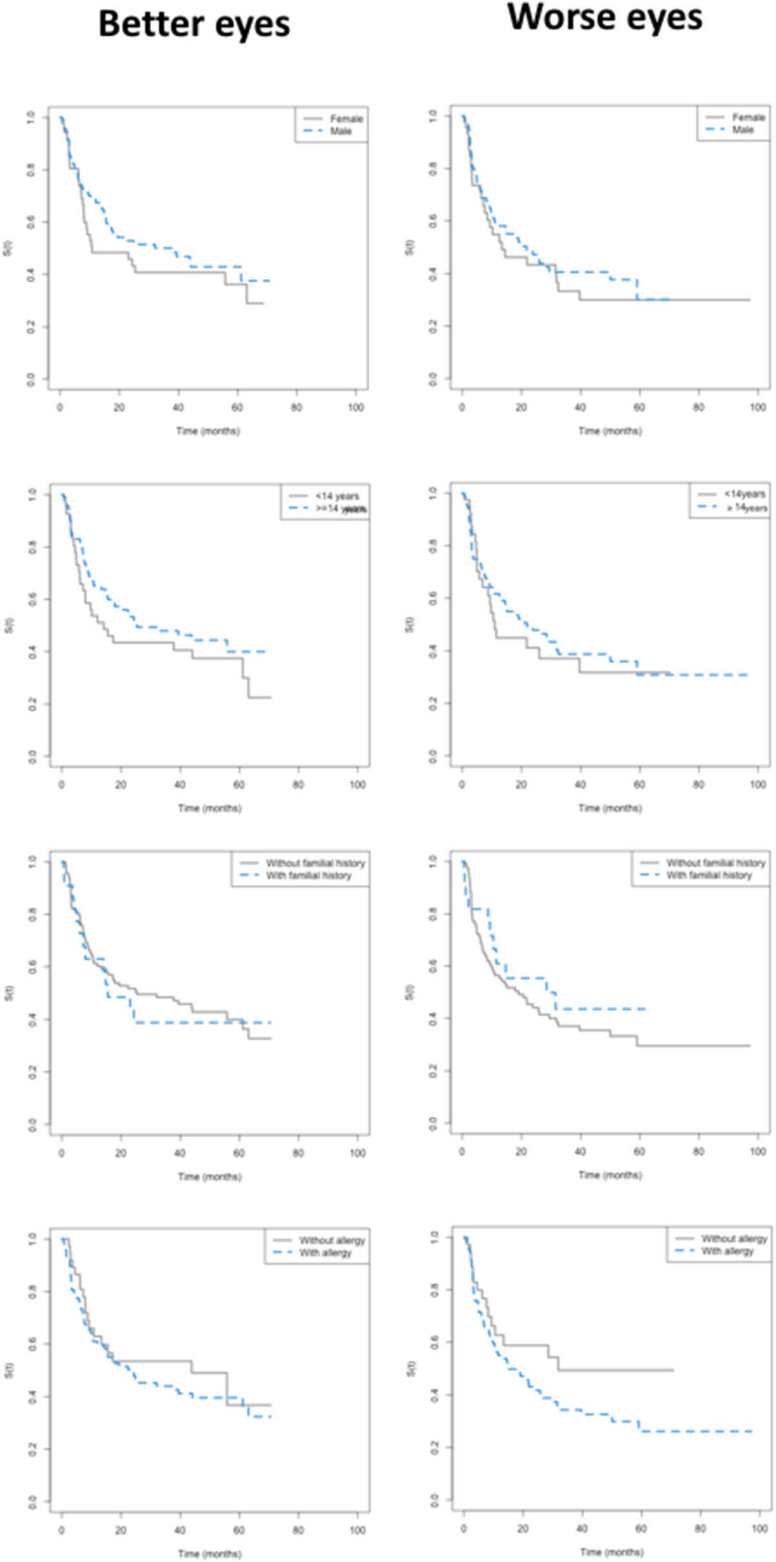
Better and worse eyes: Kaplan Meyer empirical curves of time until progression, in months, by sex, age, familial history of keratoconus, and the presence of allergy.

**Figure 6:**
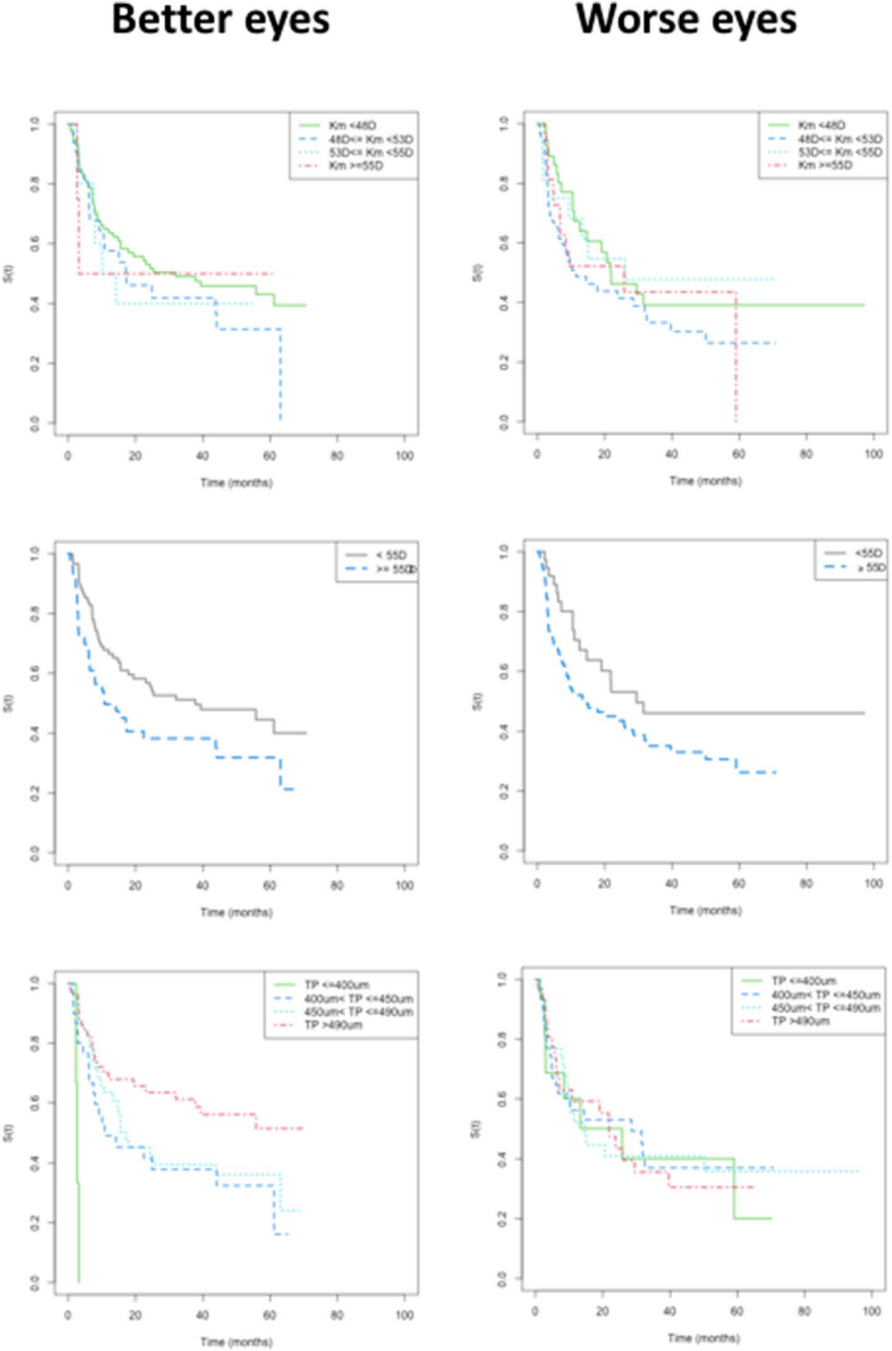
Better and worse eyes: Kaplan Meyer empirical curves of time until progression, in months, by Km, (divided into four groups^18^), by Kmax severity (< or ≥ 55D), and by thinnest pachymetry (TP) (divided into four groups^18^).

Better and worse eyes’ survival times did not differ by sex, age, familial history of keratoconus, or the presence of allergy. The survival times did not differ by Km. Both BE and WE survival times were smaller for Kmax ≥ 55 D (*p* values = 0.032 and 0.044, respectively). It was not possible to compare the group of eyes with TP ≤ 400 μm (despite the *p* value < 0.05) with the others due to the small number of individuals in this group (n = 3). Figures 5 and 6 show the survival curves, and Table 2 summarizes statistical data.

**Table 2:** Better and Worse eyes: Median time and 95% Confidence Intervals for keratoconus progression in a cohort study where 305 eyes of 168 individuals were investigated for predictors of keratoconus progression.

		Better eyes (n = 136) Median (95%CI)	*P* value	Worse eyes (n = 136) Median (95%CI)	*P* value
Sex	Female	10.8 (7.97;-)	0.323	13.4 (8.45;-)	0.512
	Male	37.8 (15.55;-)		21.8 (10.98;-)	
Age	< 14 y	14.2 (7.9;-)	0.219	11.0 (8.75;-)	0.666
	≥ 14 y	25.4 (17.1;-)		21.9 (13.4; 50)	
Familial history	No	25.4 (15.1; 63.1)	0.821	19.0 (10.2; 32.4)	0.362
	Yes	15.5 (7.9;-)		28.5 (10.5;-)	
Allergy	No	43.9 (10.8;-)	0.458	31.8 (10.5;-)	0.120
	Yes	23.1 (14.2; 61.1)		15.1 (10.2; 29.4)	
Km (D)	< 48	32.07 (15.53;-)	0.517	21.8 (12.53;-)	0.358
	≥ 48–< 53	17.12 (9.20;-)		11.5 (6.42; 39.7)	
	≥ 53–< 55	12.12 (7.58;-)		26.00 (9.37;-)	
	≥ 55	3.13 (2.67;-)		25.8 (6.87;-)	
Kmax (D)	< 55	37.8 (17.85;-)	0.032	29.4 (14.68;-)	0.044
	≥ 55	10.8 (6.42;-)		14.5 (8.75; 31.8)	
TP (μm)	≤ 400	2.67 (2.38;-)	0.014	25.8 (3.03;-)	0.965
	> 400 ≤ 450	10.82 (7.58;-)		28.5 (6.65;-)	
	> 450 ≤ 490	17.12 (13.37;-)		14.7 (9.67;-)	
	> 490	- (32.07)		21.9 (7.10;-)	
Total		24.9 (15.5; 1.1)		19.0 (11.0; 31.8)	0.301

*TP* Thinnest pachymetry.

## Discussion

The definition of keratoconus progression is still a great challenge^7^. Many parameters have been used to define progression, but the increase of one diopter or more in the maximum keratometry is the most frequent index to define keratoconus progression^21,22^. This value is based on the repeatability limits of 0.8 D observed in the Pentacam HR, using the principle of Scheimpflug (Pentacam HR, Oculus Optikgeräte GmbH, Wetzlar, Germany), in an adult cohort study with normal healthy eyes^23^. Since repeatability may vary depending on the severity of the disease^24,25^, and that there are no repeatability studies exclusive to the pediatric population^26–31^, we decided on a more conservative approach. We used a Kmax increase of 1.5 D^32^ as the event.

The present work reveals that the major factors associated with progression were: Kmax ≥ 55 D (for LE, BE, and WE), and presence of allergy (for RE).

Concerning demographic and medical data, our sample follows other authors, showing a greater proportion (2:1) of males^8,33,34^. In relation to 15% of the individuals having reported a familial history of keratoconus, this agrees with the literature that describes a prevalence of 5–19%^8,35–37^. In our sample, 70% presented with allergy (ocular allergy, rhinitis, asthma, or atopic dermatitis), as Zadnik and Rabinovitz^37,38^ observed that 44–52.9% had hay fever or allergy, and 14.9% had asthma. Contrary to Tuft et al.^39^, who observed that the RE were the worst affected, in the group BE/WE eyes, we observed that the LE were the ones with the most severe impairment in 53.7% of the patients.

Our data shows that 50% of the eyes had the event in approximately 20 months. For 12 months, the event occurred in approximately 40% of the patients, and our sample had a median Kmax (interquartile ranges) of 56.4 D (51.4–57.1). Tellouck et al.^40^ studied 109 eyes of 55 patients and described approximately 4 times less progression (11% of the eyes), defined as an increase in 1 D in Kmax. However, they evaluated older patients (age of 26.4 years) with milder disease (mean Kmax of 50 D) at one year. Fujimoto^5^ studied 217 eyes of 113 patients and pointed to a 42% progression rate in a sample with a mean Kmax ≥ 53 D in older patients (< 30 years), with a longer follow-up period, from 1.31 to 5.79 years. Chatzis and Hafezi^4^ reported 88% of 1D progression in Kmax in a sample of 59 eyes of 42 children with a mean age of 16.6 years; however, this data belongs to a referral center for corneal CXL.

Concerning BE and WE, the similar survival times in the first ten months may be due to 47 (35%) patients having both eyes with severe disease (Kmax ≥ 55 D). Meyer et al. observed a higher rate of bilateral progression if at least one of the eyes had severe keratoconus^41^. In our cohort, despite the Kmax difference between BE and WE being approximately 7 D, the survival curves were not different after 36 months. In addition to the disease being severe bilaterally in 35%, the sample size or an insufficient follow-up time may explain the lack of difference between BE and WE survival curves. Other authors^39^ have described smaller survival times for corneal transplant for the WE, while milder diseases (Kmax ≤ 53 D) take a longer time to detect progression, especially when changes in mean keratometry are used as the event^42^. Choi et al.^42^ point out that even in the milder forms, the cases that progressed had thinner corneas. Lin et al.^34^ also described changes in milder cases in patients younger than 17 years; however, they observed a borderline significant progression after 3.16 years.

Regarding sex, we observed no differences in survival times neither in RE/LE nor in BE/WE, as other authors^22,39^.

Relative to age, our data does not show different survival curves between two pediatric age groups: patients < or ≥ 14 years old. Ferdi et al. studied all group ages in a meta-analysis. They observed that patients younger than 17 years old progress more aggressively: for every 10-year increase in age, there was less 0.8 D Kmax steeping in 12 months^33^. They described a negative correlation between change in Kmax and age using data from the control group, and this points to a greater progression in younger people. Other authors also observed greater progression (shorter time for transplant) in patients aged 18 years old or younger^39^. Fujimoto et al.^5^ observed significant annual changes in the posterior curvature in younger patients, as Tellouck et al.^40^; however, they did not observe any relationship between Kmax and age. Choi et al. observed progression even in milder cases with a mean age of 21.5 years^42^, while Or et al. did not, despite the follow-up of five years, in eyes with a baseline mean Kmax of 49.6 D younger than 18 years^43^.

As for familial history, we did not find any differences between having or not having relatives with the disease, and this is in agreement with other authors^39^.

Regarding allergy, our data showed that the RE with allergy progressed more rapidly. However, this is not a consensus. Choi et al.^42^ did not find a greater frequency of atopy in the progression keratoconus group, and nor did Tuft et al.^39^ even with the presence of giant papillary conjunctivitis. One concern is that terms such as atopy, allergy, and eye rubbing may be used as synonyms when they are not. In a recent systematic review^44^, the authors showed that the predictors “allergy” and “eye rubbing” are risk factors for the presence of keratoconus, while the presence of “atopy” is not. In our sample, we considered having allergy patients with a history of at least one of the following items: allergic conjunctivitis, rhinitis, asthma, and atopic dermatitis (see frequency in Supplemental Fig. 1). We do not have information about hand dominance. Published data do not show a relationship between keratoconus laterality and hand dominance^45^, or it appears only in cases of severe rubbing^46^.

Concerning the predictor Kmax for disease progression, our data show significantly smaller survival curves for eyes with Kmax ≥ 55 D in the LE, BE, and WE. The literature shows that keratoconus progression is more likely to occur in patients with greater Kmax. In less severe forms of the disease, with mean Kmax varying from 46.97 to 49.6, the authors did not observe changes in the untreated eyes at five years^43^, or it took a longer time (7 years) to detect a 2.5 D progression^47^. In more severe cases (mean apex keratometry of 61.3), however, the authors observed a mean increase of 2.9 D at one year^48^. Fujimoto et al. also observed greater decreases in posterior curvatures in eyes with steeper posterior curvatures (more severe eyes)^5^, and showed that the annual changes in posterior best-fit sphere corneal curvature and TP were significantly higher in younger patients and patients with higher Kmax, and also pointed out that both Kmax and TP changed much faster (10 times greater) in the eyes that suffered corneal hydrops. Interestingly, in our cohort, the two eyes that had the event by acute hydrops had both Kmax of 64 D and TP of 399 and 342 μm. In older patients (mean of 25.8 years with baseline Kmax of 51.18 D) Wittig-Silva et al. observed Kmax progression after a longer period (increase in 1.7 D after 24 months)^22^. Tuft et al.^39^ studied time to corneal transplant and observed that both the minimum and maximum radius of curvature had significant effects. That is, the smaller the radius of curvature, the smaller the survival times. In a meta-analysis including 12 studies and 11,529 eyes, Ferdi et al.^33^ described a 0.7 D Kmax increase at 12 months, significantly associated with baseline Kmax and age.

Concerning pachymetry, due to a small number of patients with TP ≤ 400 μm^18^, it was impossible to build a real survival curve. Choi et al. show that progression is more likely to occur in thinner corneas, even in mild cases^42^. Data from Wittig-Silva et al.^49^ reinforces that pachymetric thinning is associated with Kmax increase even in older patients. In addition, Or et al.^43^ observed no changes in Kmax or TP in thicker corneas of control untreated eyes of a pediatric group. This points to concomitant changes in both Kmax and TP. It is important to point out that thinning is greater in younger patients with greater disease severity or before hydrops^5^, and in thinner corneas^6^. A recent model to predict keratoconus progression, using survival curves in a cohort of patients with a mean age of 28.28 years, explains 33.3% of the variation in time to event. Age and Kmax are the main predictors. TP is a predictor with a much smaller contribution. In a sensitivity analysis, the survival probabilities at five years are 40 and 27% for the best and worst cases, respectively^50^.

Our study has some limitations. The censored patients due to CXL procedures may have generated some bias towards no progression. However, among the 13 patients censored due to CXL, 11 had a Kmax progression that was less than 1.0 D, and 2 had progression between 1.0 and 1.3 D. Although the study evaluated all the patients that were attended in this corneal ambulatory with a minimum follow-up of 36 months, there was a loss of 25 patients who attended only the first consultation and did not return for follow-up, and this represents a loss of approximately 12% of the total cohort sample size. We did not look for other tomographic predictors of keratoconus progression, such as changes in posterior curvature or vertical coma, that occur earlier than changes in the anterior keratometry^40^.

In conclusion, in our pediatric cohort, different pediatric group ages were not different for progression. Our data reinforce that the steepest corneas are associated with faster progression and therefore need close follow-up. The presence of allergy is also a predictor of keratoconus progression.

## Supplementary Information

Supplementary Information 1.

Supplementary Information 2.

Supplementary Information 3.

Supplementary Information 4.

Supplementary Information 5.
