# Supplementary figures and images for: Natural history and predictors for progression in pediatric keratoconus
Source: Sci Rep. 2023 Mar 27;13:4940. doi: 10.1038/s41598-023-32176-5 (PMC10042985; doi:10.1038/s41598-023-32176-5)

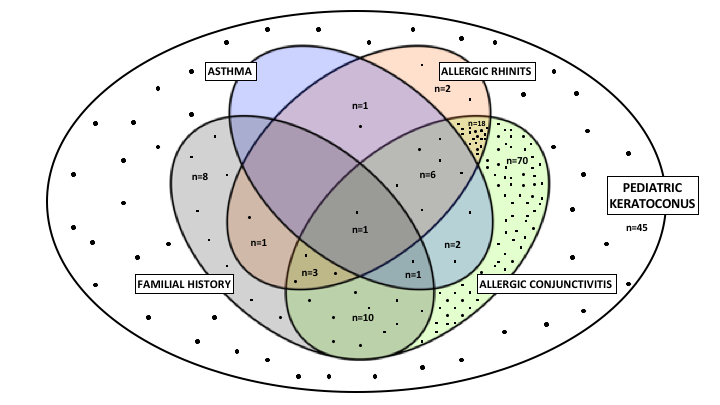

Supplement: Supplementary file 1 — Supplementary Information 1. [file 41598_2023_32176_MOESM1_ESM.tiff]
