# Supplementary material for: Natural history and predictors for progression in pediatric keratoconus
Source: Sci Rep. 2023 Mar 27;13:4940. doi: 10.1038/s41598-023-32176-5 (PMC10042985; doi:10.1038/s41598-023-32176-5)
Supplement: Supplementary file 2 — Supplementary Information 2. [file 41598_2023_32176_MOESM2_ESM.docx]

Supplemental Table 1. Pediatric keratoconus, right and left eyes: tomographic data according to ABCD grading system.

| N=305 |  | N | Right | Left |
| --- | --- | --- | --- | --- |
| Km (D) | < 48 | 134 (44%) | 69 (44%) | 65 (44%) |
|  | ≥48 - < 53 | 97 (32%) | 48 (31%) | 49 (33%) |
|  | ≥53 - < 55 | 30 (10%) | 17 (11%) | 13 (9%) |
|  | ≥ 55 | 44 (14%) | 22 (14%) | 22 (15%) |
| Kmax (D) | < 55 | 132 (43%) | 68 (44%) | 64 (43%) |
|  | ≥55 | 173 (57%) | 88 (56%) | 85 (57%) |
| Thinnest Pachymetry | ≤400 | 27 (9%) | 16 (11%) | 11 (7%) |
|  | >400 - ≤450 | 86 (28%) | 40 (26%) | 46 (31%) |
|  | >450 - ≤490 | 93 (30%) | 49 (31%) | 44 (30%) |
|  | >490 | 90 (30%) | 48 (31%) | 42 (28%) |
