# Supplementary material for: Natural history and predictors for progression in pediatric keratoconus
Source: Sci Rep. 2023 Mar 27;13:4940. doi: 10.1038/s41598-023-32176-5 (PMC10042985; doi:10.1038/s41598-023-32176-5)
Supplement: Supplementary file 3 — Supplementary Information 3. [file 41598_2023_32176_MOESM3_ESM.docx]

Supplemental Table 2. Pediatric keratoconus, better and worse eyes: tomographic data according to ABCD grading system.

| N=272 |  | N | Better | Worse |
| --- | --- | --- | --- | --- |
| Km (D) | < 48 | 127 (47%) | 90 (66%) | 37 (27%) |
|  | ≥48 - < 53 | 87 (32%) | 32 (24%) | 55 (40%) |
|  | ≥53 - < 55 | 27 (10%) | 10 (7%) | 17 (13%) |
|  | ≥ 55 | 31 (11%) | 4 (3%) | 27 (20%) |
| Kmax (D) | < 55 | 127 (47%) | 89 (65%) | 37 (27%) |
|  | ≥55 | 145 (53%) | 47 (35%) | 99 73%) |
| Thinnest Pachymetry | ≤400 | 17 (6%) | 3 (2%) | 16 (12%) |
|  | >400 - ≤450 | 76 (28%) | 30 (23%) | 45 (34%) |
|  | >450 - ≤490 | 81 (30%) | 43 (32%) | 39 (30%) |
|  | >490 | 90 (33%) | 57 (43%) | 31 (24%) |
