# Supplementary material for: Natural history and predictors for progression in pediatric keratoconus
Source: Sci Rep. 2023 Mar 27;13:4940. doi: 10.1038/s41598-023-32176-5 (PMC10042985; doi:10.1038/s41598-023-32176-5)
Supplement: Supplementary file 4 — Supplementary Information 4. [file 41598_2023_32176_MOESM4_ESM.docx]

Supplemental Table 3. Descriptive measures of the mean and the maximum keratometry and presenting visual acuity for the right, left, better, and worse eyes, and the total sample.

| **Group** | **Variable** | **N** | **Min** | **Máx** | **Q1** | **Median** | **Mean** | **Q3** | **SD** |
| --- | --- | --- | --- | --- | --- | --- | --- | --- | --- |
| **RE** | Km (D) | 156 | 41.8 | 69.8 | 45.9 | 48.3 | 49.6 | 52.9 | 5.2 |
|  | KMax (D) | 156 | 43.6 | 80.6 | 52.4 | 57.1 | 57.3 | 62.2 | 7.8 |
|  | TP | 153 | 320 | 555 | 435 | 470 | 462.5 | 498 | 46.2 |
|  | VA | 156 | 0.0 | 1.1 | 0.1 | 0.3 | 0.3 | 0.5 | 0.3 |
| **LE** | Km (D) | 149 | 42.0 | 63.9 | 45.8 | 48.8 | 49.5 | 52.6 | 4.9 |
|  | KMax (D) | 149 | 43.5 | 79.5 | 51.6 | 56.2 | 57.0 | 62.2 | 7.7 |
|  | TP | 143 | 349 | 582 | 431 | 465 | 465.7 | 502.5 | 48.7 |
|  | VA | 146 | 0.0 | 1.1 | 0.1 | 0.3 | 0.3 | 0.48 | 0.37 |
| **BE** | Km (D) | 136 | 41.8 | 60.50 | 44.0 | 46.5 | 47.1 | 49.0 | 3.9 |
|  | KMax (D) | 136 | 43.5 | 68.4 | 47.5 | 52.2 | 53.0 | 57.2 | 6.4 |
|  | TP | 133 | 373 | 582 | 454 | 484 | 482.3 | 512 | 42.0 |
|  | VA | 134 | 0 | 0.7 | 0 | 0.15 | 0.19 | 0.3 | 0.2 |
| **WE** | Km (D) | 136 | 43.4 | 69.8 | 47.8 | 50.1 | 51.1 | 54.0 | 4.9 |
|  | KMax (D) | 136 | 45.6 | 80.6 | 54.8 | 58.9 | 60.1 | 64.7 | 6.8 |
|  | TP | 131 | 320 | 555 | 421.5 | 453 | 453.5 | 489 | 46.2 |
|  | VA | 135 | 0 | 1.1 | 0.2 | 0.40 | 0.42 | 0.6 | 0.3 |
| **Total** | Km (D) | 305 | 41.8 | 69.8 | 45.8 | 48.5 | 49.5 | 52.7 | 5.1 |
|  | KMax (D) | 305 | 43.5 | 80.6 | 51.4 | 56.4 | 57.1 | 80.6 | 7.8 |
|  | TP | 296 | 320 | 582 | 431 | 467 | 464 | 499 | 47.4 |
|  | VA | 302 | 0.0 | 1.1 | 0.1 | 0.3 | 0.32 | 0.5 | 0.3 |

RE: right eyes; LE: left eyes; BE: better eyes; WE: worse eyes; Km: mean keratometry, Kmax: maximum keratometry; TP: thinnest pachymetry; VA: presenting visual acuity (logMAR) when the patient entered the study.
