# Supplementary material for: Natural history and predictors for progression in pediatric keratoconus
Source: Sci Rep. 2023 Mar 27;13:4940. doi: 10.1038/s41598-023-32176-5 (PMC10042985; doi:10.1038/s41598-023-32176-5)
Supplement: Supplementary file 5 — Supplementary Information 5. [file 41598_2023_32176_MOESM5_ESM.docx]

Supplemental Table 4. Right and left eyes: Median time and 95% Confidence Intervals for keratoconus progression in a cohort study where 305 eyes of 168 individuals were investigated for predictors of keratoconus progression.

|  |  | **Right eyes (n=156)**  **Median (95%CI)** | **P-value** | **Left eyes (n=149)**  **Median (95%CI)** | **P-value** |
| --- | --- | --- | --- | --- | --- |
| Sex | Female  Male | 14.5 (8.8;-)  25.8 (15.1;59.1) | 0.868 | 13.4 (7.9;55.8)  24.9 (17.4;-) | 0.152 |
| Age | <14 y  ≥ 14 y | 24.6 (6.87;-)  22.2 (14.8;45.2) | 0.965 | 10.6 (7.9;44.1)  32.4 (19.4;-) | 0.0653 |
| Familial history | no  yes | 23.8 (15.07;45.2)  15.2 (9.37;-) | 0.717 | 24.9 (12.5;50.0)  19.0 (10.5;-) | 0.813 |
| Allergy | no  yes | - (17.12;-)  15.5 (9.67;32.1) | **0.0318** | 32.5 (10.5;-)  21.8 (14.2;50) | 0.846 |
| Km (D) | < 48  ≥48 - < 53  ≥53 - < 55  ≥ 55 | 31.6 (17.85;-)  14.5 (6.18;43.9)  15.1 (7.58;-)  25.8 (6.87;-) | 0.458 | 22.4 (12.03;-)  31.83 (10.82;-)  26.00 (13.40;-)  9.28 (4.83;-) | 0.612 |
| Kmax (D) | < 55  ≥ 55 | 32.1 (17.85;-)  14.5 (7.97;43.9) | 0.129 | 25.4 (19.0;-)  17.8 (10.1;44.1) | 0.0428 |
| TP | ≤400  >400 - ≤450  >450 -≤490  >490 | 8.45 (2.68,-)  12.42 (7.58,-)  17.12 (14.80;-)  37.78 (21.87;-) | 0.779 | 13.4 (3.13;-)  22.4 (10.08;-)  24.4 (10.50;-)  55.8 (19.40;-) | 0.337 |
| Total |  | 21.9 (14.5;39.7) | 0.76 | (13.4;50.0) | 0.76 |

TP: thinnest pachymetry
